# ‘Eating with Others’: planning, developing and optimising a self-management intervention to promote social eating for patients living with and beyond head and neck cancer

**DOI:** 10.1007/s00520-024-09083-0

**Published:** 2024-12-16

**Authors:** D. M. Dornan, C. J. Semple, A. Moorhead

**Affiliations:** 1 School of Nursing and Midwifery, Queen’s University, Belfast, UK; 2 School of Nursing, Institute of Nursing and Health Research, Ulster University, Newtownabbey, UK & Cancer Services, South Eastern Health and Social Care Trust, Belfast, UK; 3 School of Communication and Media, Institute of Nursing and Health Research, Ulster University, Newtownabbey, UK

**Keywords:** Head and neck cancer, Social, Eating, Commensality, Survivorship, Self-management, Intervention development

## Abstract

**Introduction:**

After treatment for head and neck cancer (HNC), up to 90% of patients have difficulties eating and drinking. Despite the enormity of challenges explicitly relating to the social dimension of eating, there are limited extant interventions to specifically support social eating, nor any replicable for use in contemporary clinical practice. This study aims to plan, develop and optimise a self-management intervention to promote social eating for patients living with and beyond HNC.

**Methods:**

This research was intervention development of a self-management ‘Eating with Others’ resource, guided by the person-based approach (PBA) framework. Initially, a systematic review was conducted, with 24 included studies exploring HNC patients’ social eating experiences, followed by thematically analysed qualitative interviews with patients (*n* = 14), family members (*n* = 12) and healthcare professionals (*n* = 13). Alongside this data, iterative input was sought from an advisory group (*n* = 22) to culminate in an intervention prototype. The intervention prototype was iteratively user-tested over three cycles for usability and acceptability, using think-aloud interviews (*n* = 10).

**Results:**

A patient-centred, evidence-based and theory-driven self-management resource, entitled ‘Eating with Others’, was designed to promote social eating for patients with HNC. Sections included the benefits of social eating; the impact of HNC on social eating, strategies and reflective activities to overcome social eating barriers; and the use of a social eating card for restaurants. The think-aloud interviews revealed that the resource was appropriate and acceptable for patients with HNC.

**Conclusion:**

The systematic and iterative PBA intervention development framework enabled empirical research findings, relevant theory and extensive advisory group involvement to design an acceptable self-management social eating intervention for patients living with and beyond HNC. Mixed-methods evaluation is required to determine feasibility in clinical practice.

## Introduction

Social eating is a fundamental aspect of human life that shapes culture, daily routines and identity, as individuals often define their sense of self through social interactions, roles within groups and the use of food and drink as a form of self-expression and enjoyment [1, 2]. Up to 90% of patients living with and beyond (LWB) head and neck cancer (HNC) experience eating and drinking changes, leading to physical, emotional and social losses and a changed meaning of food [3]. The social dimension of eating is a key post-treatment challenge [4], with a recent study showing that > 63% of patients had social eating issues 12 months post-treatment [5]. The chronicity of the problem and loss, contributing to a sense of grief, indicates the need for ongoing supportive care [6]. Whilst quantitative data indicates the significance of social eating challenges, quality of life (QOL) measurements, even those specific to HNC, fail to capture the subjective meaning and impact of ‘trouble with social eating’ on everyday life [5, 7]. Qualitative studies show that social eating affects family togetherness, leading to separate mealtimes and reduced social occasions [8–10], causing restricted social lives for patients and family members (FM) and contributing to a sense of grief [11, 12]. Alongside this, HNC incidences are rising, particularly HPV-related HNCs, with improved long-term survival rates [13, 14].

Despite recognition of the ongoing challenge of social eating, there are few supportive interventions for patients, families, or education for healthcare professionals (HCP). Existing interventions like NUTRI-HAB [15] have not focused on the social dimension of eating [16] or lack generalisability to routine care due to specific HCP training requirements [6, 17].

Current literature shows the interconnectedness of physical, psychological and social recovery for patients LWB HNC [18, 19]. Given the impact of social eating challenges on patients and families, a social eating intervention developed through a biopsychosocial lens is needed [20, 21]. Dunne et al. [22, 23] emphasised the benefits of self-management strategies for post-treatment coping. Therefore, this paper aims to demonstrate the planning, development and optimisation of the theory-driven, evidence-based ‘Eating with Others’ self-management intervention to promote social eating for patients LWB HNC.

## Methods

### Intervention development framework

The research design, guided by the person-based approach (PBA) intervention development framework [24], planned, designed and optimised the ‘Eating with Others’ self-management intervention. The PBA has a central focus on qualitative evidence and emphasises the integral input from patient and public involvement (PPI), especially end-users, across all intervention development stages [25]. This paper details Phase 1: Intervention Planning and Phase 2: Intervention Optimisation, with Phase 3: Intervention Evaluation (mixed methods) determined as part of a subsequent study. Whilst Phase 1 data (systematic review and qualitative interviews) have been published in detail elsewhere, they are briefly outlined below to provide context.

### Theory-driven intervention

The ‘Eating with Others’ self-management intervention required multiple theoretical frameworks due to the complex nature of social eating challenges [12, 18]. The biopsychosocial model [26] was used to frame the content development, reflecting the established need for post-treatment supportive care in HNC survivorship literature. Additionally, self-management theory was utilised to empower individuals to manage their chronic disease [27, 28]. The Individual and Family Self-Management Theory (IFSMT) [29] was chosen for its emphasis on the role of family members in supporting HNC patients [30], addressing gaps in previous self-management research by encouraging family engagement for successful behaviour change [31, 32].

The paper will sequentially present Phase 1: Intervention Planning (three parts) followed by Phase 2: Intervention Optimisation (three parts). Integrating methods and results for each standalone part, sequentially, will aid flow and clarity for the reporting on planning, developing and optimisation of this complex healthcare intervention, ‘Eating with Others’.

### Phase 1: Intervention Planning of ‘Eating with Others’

As guided by PBA, Phase 1: Intervention Planning had three components: Part (1) review of literature [33], Part (2) qualitative research [34, 35] and Part (3) the development of guiding principles.

#### Phase 1, Part 1: review of literature

A qualitative systematic review was conducted to understand the social eating experiences of patients LWB HNC and is reported in full elsewhere [33]. Findings from the review highlighted a lack of available resources to support patients with social eating challenges, whilst simultaneously identifying FMs as key support providers for patients with HNC but may have their own unique challenges surrounding social eating.

#### Phase 1, Part 2: qualitative research

##### Sampling, recruitment and data collection

Given the inherent gaps in the literature and guided by the PBA [24], qualitative research with three sample populations was conducted, including patients, FMs and HCPs. The full methods and findings from the qualitative data with patients and FM are reported in published studies elsewhere [33, 34]. Findings from patient data (*n* = 14) revealed that social eating became a conscious process, with patients being mindful of food and their appearance to others. Strategies to enhance social eating included minimising attention on eating, managing expectations and receiving support from others, as detailed in another publication [34]. FM data (*n* = 12) highlighted the challenges both parties faced and the efforts by FMs to support social eating togetherness. FMs often advocated in restaurants or suggested alternative arrangements to facilitate social engagement. More detailed findings from FMs are published elsewhere [35].

In addition, there was HCP recruitment open to multidisciplinary team (MDT) members: CNS, surgeons, oncologists, dietitians, speech and language therapists (SLT), nursing staff, radiographers, physiotherapists and psycho-oncology staff (Table 1). After obtaining consent, HCP data was collected via online focus groups. The HCP topic guide was developed and informed by the literature and expertise of the co-authors. Participants were recruited from three participating healthcare trusts that provide services to patients with HNC. Data collections were conducted by one Registered Nurse (MD), with experience in qualitative data collection and not known to the participants. Three focus groups (*n* = 3) with HCPs (*n* = 13) took place via an online platform. During each interview, the Researcher (MD) took notes, followed by personal, reflective notes post-interview.

**Table 1:** Participants’ inclusion and exclusion criteria for HCP focus group

Healthcare professionals inclusion	Healthcare professionals exclusion
• Registered HCP • Provided direct care to patients with HNC • Able to provide informed consent	• Non-clinical HCP • Does not hold valid healthcare registration • Healthcare students

##### Ethical approval and considerations

Ethical approval was gained from the NHS Research Ethics Committee (20/WA/0253) and research governance from local participating healthcare trusts within the UK. Informed consent was obtained prior to data collection. A distress protocol was established to minimise the risk of harm and promote non-maleficence in the research and utilisation required for one FM.

##### Data analysis

Data from the interviews were analysed using Braun and Clarke’s [36] method of reflexive thematic analysis. NVivo data management software was used to support the organisation of the data where the three data sets were analysed separately. Credibility, transferability and dependability were considered to promote rigour in qualitative data analysis [37]. Demographic data from the interviews was summarised using descriptive statistics.

##### Findings

Patient (*n* = 14) and FM (*n* = 12) data revealed that social eating is a conscious effort requiring strategies and support, with detailed findings published elsewhere [34, 35]. Whilst both patients and FMs described the limited interactions and support from HCPs concerning social eating, however, some HCPs from the focus group data demonstrated an awareness of patient and FM challenges around social eating, offering a potential role in providing professional support. HCPs from the community offer critical insights into their distinct approaches to providing social eating support, which differ from those employed by acute care HCPs. This distinction highlights the continuous support for social eating throughout the cancer trajectory.

#### Phase 1, Part 3: guiding principles for ‘Eating with Others’

Guiding principles are a vital component of PBA providing the blueprint of the key content, informed by identifying the key: (i) issues, (ii) design objectives and (iii) design features of the intervention [24]. The guiding principles for ‘Eating with Others’ were aligned with findings from the systematic review and qualitative data derived from the three study populations and guided by the biopsychosocial model [26] and the IFSMT [29] (see Table 2). The Key issues were identified through a synthesis of the collected empirical data and literature led by the research team (authors), alongside input from an expert group to include PPI.

**Table 2:** Guiding principles

Key issue (the problem)	Key design objectives (what is the intervention going to do about the problem)	Key design features (how is the intervention going to do this)
Patients and FMs report a lack of understanding and awareness of the challenges of social eating living with HNC with a lack of resources to provide support	Increase knowledge of social eating to better understand the challenges of eating with others after HNC treatment for patients and families	• Provide an accessible, evidence-based resource that addresses the key issues of social eating relevant to patients living with HNC and their families [33–35] • In clear and understandable language, outline the meaning of social eating [33] • Emphasise the biopsychosocial benefits of eating with others for patient and family well-being [33] • Recognise the impact of HNC treatment’s side effects on the physical, functional, psychological and social aspects of eating [26, 33, 34] • Highlight the losses associated with social eating due to HNC treatment [33]
Social eating is an ongoing challenge that presents at different times. However, early intervention can improve social eating engagement and enjoyment	Promote early integration of social eating post-treatment for HNC, focusing on patient-centred self-management skills and support from family	• A family-centred resource that uses inclusive language to engage both the patient with HNC and FMs about social eating • A resource guided by IFSMT [29] • A resource that is accessible to patients at any time through an online platform of survivorship resources • The resource will recommend early engagement to promote confidence and self-esteem/efficacy to reduce avoidance of social eating later in recovery [29] • Signpost to HCPs and appropriate organisations where patients and FMs can access additional support
Social eating is more important to some patients than others, as patients and FMs have different life circumstances, preferences and social networks	Identify the context in which patients socially eat, the opportunity to engage with this resource and who can support them with social eating	• Identify how treatment for HNC has impacted the patient and family • Identify where they ate with others before treatment and how that has changed • Identify one’s role and identify associated with social eating, including FMs and thinking broadly about how they are included in social eating • Acknowledge how social eating may change over the treatment and recovery period, but early engagement with this resource is essential
Social eating is a conscious process after HNC treatment where people become conscious of food, their functionality and how it appears to others [34]	Promote confidence in social eating by increasing self-efficacy [29]	• Following Bandura’s theory of self-efficacy to promote confidence in capabilities and beliefs about function and ability [29] • Mastery experience—building up small success: trial and error approach to social eating, examples of step approach, graded exposure and SMART goals • Social modelling—strengthening self-beliefs, providing stories of other patients and FMs stories in the resource and supporting video [29] • Social persuasion—opportunity to talk about this with FMs and HCPs. Providing verbal encouragement to support social eating. Acknowledging that it can be a challenge, but they are not alone • Psychological responses—minimising potential stress by providing examples of practical strategies of ways to cope with SE that have been identified in the literature review and research interviews

### Phase 2: Intervention optimisation for ‘Eating with Others’

Phase 2: Intervention Optimisation of ‘Eating with Others’ consisted of three components depicted as a cyclical process as guided by PBA: Part (1) establishing an advisory group, Part (2) iterative intervention development and Part (3) qualitative user testing of draft materials**.**

#### Phase 2, Part 1: establishing an advisory group

An advisory group of 22 members was identified due to their experience relating to HNC or intervention design. This included patients, FMs, acute and community HCPs working in medicine, nursing, dietetics, speech and language therapy, psychology and other academic and educationists. Some advisory group members were familiar with the research study due to their participation in Phase 1.

#### Phase 2, Part 2: intervention development

The initial draft content of the ‘Eating with Others’ intervention was developed using an online web application by lead author (MD) and updated following scrutiny and critique of content, title, wording, colours, phraseology and patient stories with the research team (co-authors). Following several drafts, an agreed iteration (version 2.0) was circulated to members of the advisory group for feedback using the proforma, with columns for feedback and then potential and actual changes noted. Each intervention draft was updated to reflect the input before further review (see Fig. 2).

Having harnessed extensive engagement and iterative feedback from the advisory group, the ‘Eating with Others’ version 4.1 was sent to a graphic designer, with content presented in eight sections: a brief overview of HNC, a summary of social eating and its benefits, how social eating impacts patients and family members, reflections on a social eating situation with writing exercises, tips to promote a positive social eating experience, making a personal plan, signposting for further professional support and a social eating card to use in restaurants and cafes.

**Fig. 1:**
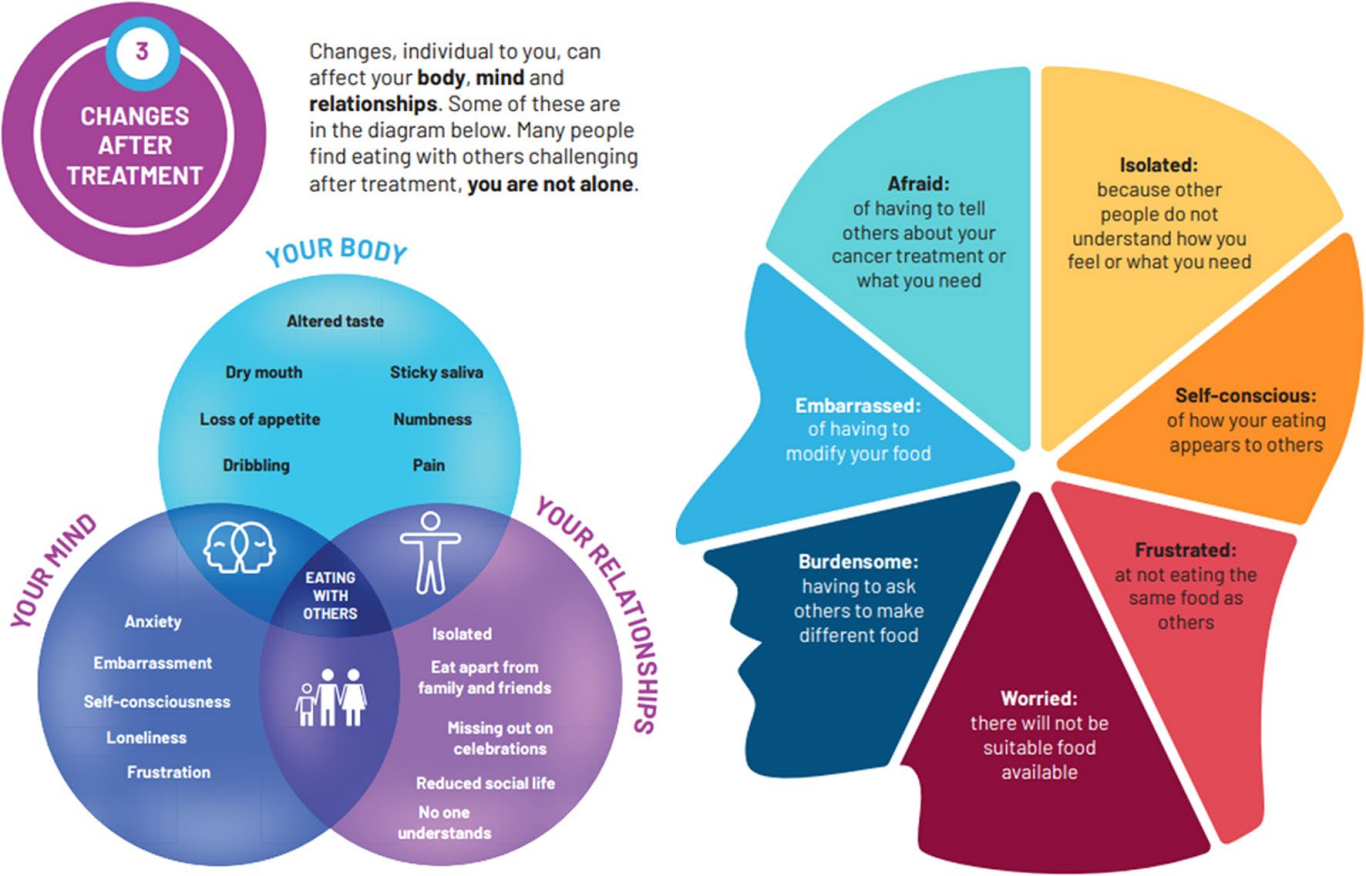
Examples of pages of intervention content

The graphic designer produced version 5.0 (Fig. 1), which was iteratively updated to reflect the guiding principles (Table 2). This subsequent version (version 6.0) was considered an evidence-based, theory-driven ‘Eating with Others’ intervention prototype ready for user testing.

#### Phase 2, Part 3: user testing of ‘Eating with Others’ using think-aloud interviews

Think-aloud interviews were selected for this aspect of the research, being commonly used to determine acceptability and comprehension of technical language with target groups by participants’ verbalising thoughts whilst completing a task [38]. As advocated within PBA, think-aloud interviews also promote optimisation by ensuring the intervention is appropriate, relevant and applicable whilst providing opportunities for feedback on potential behaviour change [39].

##### Sampling, recruitment and data collection

**Fig. 2:**
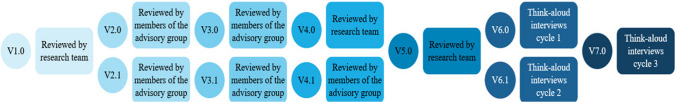
Overview of drafting and refining of intervention. Footnote: versions 0.0 and 0.1—the difference between a 0.0 and 0.1 reflects minor changes only

Patients and HCPs were recruited for think-aloud interviews using the same eligibility criteria as Phase 1 (Table 3). An iterative approach to data collection and analysis was utilised for user testing to enact change to the intervention prototype, totalling three cycles of iterative refinement and re-testing (Fig. 2). Following informed consent, data for think-aloud interviews were collected by video call (*n* = 4), telephone (*n* = 3) and face-to-face (*n* = 3), with five patients and five HCPs, from three participating healthcare trusts, with interviews lasting between 30 and 45 min. Some participants were familiar with the study and had participated in Phase 1. Prompts were used to guide the process to promote consistency and rigour in the process [40]. The think-aloud question guide was flexible to focus on areas where participants had more feedback but ensured the entire document was considered page by page. During the think-aloud interviews, participants were asked about the layout, content and general concepts of the intervention. Interviews were audio-recorded and field notes taken during the interview, with reflective notes made after the interview. A summary of participant characteristics is displayed in Table 4.

**Table 3:** Participants’ inclusion and exclusion criteria for patients and HCP think-aloud interviews

Patient inclusion	Patient exclusion
• Age > 18 years old • Diagnosis of head and neck cancer • Completed treatment of curative intent for HNC • Post-treatment challenges with eating and drinking • Able to provide informed consent • Be able to communicate in English	• Receiving treatment for recurrence • Receiving palliative or end-of-life treatment • Treatment not yet completed • Dementia or cognitive impairment
Healthcare professionals inclusion	Healthcare professionals exclusion
• Registered HCP • Provided direct care to patients with HNC • Able to provide informed consent	• Non-clinical HCP • Does not hold valid healthcare registration • Healthcare students

**Table 4:** Think-aloud interview participant characteristics (*n* = 10)

Patient ID	Gender	Age	Tumour location	Treatment	Time since completion of treatment	Employment	Lives with
1	M	46–60	Oral cavity	Sx, Rx, Cx	1–2 years	Sick leave	Spouse and children
2	M	75 +	Sinus	Sx, Rx	1–2 years	Retired	Spouse
3	M	46–60	Oral cavity	Sx, Rx, Cx	1–2 years	Employed	Spouse and children
4	M	46–60	Oral cavity	Sx, Rx	2–5 years	Employed	Spouse
5	F	46–60	Oral cavity	Sx	1–2 years	Employed	Alone
HCP ID	Gender	Role	Setting	Experience (years)			
6	F	CNS	Acute	10–15			
7	F	CNS	Acute	1–5			
8	F	Speech and language therapist	Community	5–10			
9	F	Dietitian	Acute	10–15			
10	M	Consultant HNC surgeon	Acute	10–15			

*Sx* surgery, *Rx* radiotherapy, *Cx* chemotherapy, *CNS* clinical nurse specialist

##### Data analysis

Using an iterative approach, data analysis commenced following the first cycle of think-aloud interview [40]. Thus, the process moved between data collection, analysis, making modifications to the resource and then continuing data collection. Interviews were transcribed verbatim and using a content analysis approach data was extracted unto an extraction sheet on Microsoft Word, under the following headings: negative comments, positive comments and possible changes with supporting quotes as appropriate. The research team met after each cycle and decided what modifications should or should not be made, guided by the ‘Must have, Should have, Could have, Would like (MoSCoW) prioritisation model [40], as guided within PBA [24]. After the third cycle of think-aloud interviews, the team considered that no additional changes had been captured to impact behaviour change.

##### Findings

Overall, the feedback from initial testing of the ‘Eating with Others’ intervention using think-aloud interviews was positive. Changes in cycle one are mostly related to embedding additional strategies to promote confidence in eating with others, as well as emphasising the enjoyment often obtained when eating with others, themed as ‘Positive integration of behavioural and cognitive components’. Cycle two is predominately related to clarity on language and terminology used throughout the resource, themed as ‘Appropriateness of terminology and language’. Cycle three affirmed changes that were made to the resource prototype during earlier user-testing cycles were acceptable. During cycle three, a few additional minor suggestions were adopted such as removing redundant words and repositioning of text to aid flow, themed as ‘Layout and presentation’. Findings from the ten think-aloud interviews are discussed under these three broad themes below.

##### Theme one: positive integration of behavioural and cognitive components

Overall, it was remarked by both patients and professionals that this intervention normalised the social eating challenges experienced by patients LWB HNC, with Participant 4 (patient) reporting:

it brought home for me that others have been and still are going through a similar situation…confirmation that I am not on my own. (Participant 4, patient)

Both patients and professionals considered the availability of this novel ‘Eating with Others’ intervention would enable patients to gain confidence and take a ‘huge step forward’ (Participant 1, patient), meeting a gap in clinical practice as currently there were no other resources available to promote social eating.

this will be a really great resource for us to give to patients who are having difficulties eating socially after treatment… up to now we didn’t have anything specific to give or help them…it is such an important area. (Participant 7, professional (CNS))

Several participants suggested in test cycle 1 the need to integrate additional cognitive components surrounding the enjoyment often achieved when eating with others, which was adopted and retested with positive feedback in cycle 2.

Not sure that it brings out that it is great to eat with others. Need to bring out benefits of social eating – the enjoyment of it and the enjoyment of life. (Participant 2, patient)

Furthermore, in all testing, cycle’s participants highlighted the social eating card was an excellent concept and a useful tool to promote planning and gain confidence before eating out in restaurants.

Social eating card can give an establishment prior notice or on arrival – make people feel at ease. (Participant 4, patient)

Overall, the ‘Eating with Others’ intervention was reported as a useful resource for patients LWB HNC, when planning for social eating.

Theme two: appropriateness of terminology and language.

In general, participants positively commented that the language was suitable for patients, often referred to as at the ‘right level’, ‘not confusing’ or ‘technical’.

Nonetheless, in cycles two and three, there were a few areas where terminology was modified to improve clarity and understanding. For example, one participant (professional, speech and language therapist) considered that reordering of paragraphs one and two on page 10 would enable the safety aspect of eating after HNC treatment to be ‘front and central (Participant 8, professional)’. Also, some patients and professionals felt there needed to be a clearer acknowledgement of the permanency of tube feeding for some patients; therefore, the text on page 11 was modified from ‘Some people may feel that they will never be able to eat socially again’ to ‘Some people may not be able to eat socially again and continue with tube feeding’.

Overall, the ‘Eating with Others’ resource was found to be appropriate in terminology and language.

##### Theme three: layout and presentation

Overwhelmingly, participants reported positively on the layout and presentation of the resource. Key aspects included the design, colour, use of silhouettes rather than pictures and the thoughtful use of text, therefore not overburdening patients with information during the rehabilitation phase of their treatment.

last thing you want is too much information if you are feeling miserable. (Participant 2, patient)

Thus, the ‘Eating with Others’ resource was reported to have an appropriate layout and presentation.

Overall, the think-aloud interviews found that the ‘Eating with Others’ resource was appropriate and acceptable for patients with HNC.

## Discussion

‘Eating with Others’ is a novel, theory-driven, evidence-based self-management intervention reported to be acceptable for patients. Previous commensality interventions in HNC provided some fundamental elements for social eating [6, 15–17], but lacked applicability in models of modern healthcare. ‘Eating with Others’ builds on empirical evidence [33–35], underpinned by the biopsychosocial model [26] and IFSMT [29], guided by the PBA framework [24]. It addresses post-treatment social eating challenges, personal reflections, patient stories, practical tips and goal-setting strategies and includes a social eating card for HNC patients.

Social eating involves food, individuals and context [41]. This intervention does not explicitly consider specific food items, as nutritional aspects require specialist advice and risk management [42]. Instead, it focuses on the dynamics between patients and others, primarily family and friends. The intervention emphasises the role of FMs, encouraging users to share experiences and responses with FMs. Environmental factors emerged prominently, with patients more comfortable in familiar settings [34]. Adaptability allows patients to enjoy certain restaurants or holidays by tailoring environments to their needs. The intervention encourages revisitation, reflecting the dynamic nature of commensality, unlike other interventions reliant on HCPs for dissemination [17].

Self-management resources are an expanding contemporary method of care delivery in cancer survivorship to empower individuals in managing their healthcare needs [43, 44]. Through iterative discussions with the advisory group and the empirical data garnered from patients, FMs and HCPs, self-management was deemed an appropriate method of delivering information and support for social eating for patients LWB HNC. Self-management is viewed as active coping within the context of daily life for individuals with chronic diseases [27] which aligns with the reported chronicity of social eating challenges [33, 44]. Recommendations for self-management in cancer survivors [44] were adopted within the ‘Eating with Others’ intervention, which included tailoring the intervention for this specific patient population and addressing the unique needs of individuals LWB HNC. The utilisation of self-management strategies is particularly pertinent given the challenges of heterogeneity in HNC survivorship, encompassing diverse tumour types, treatments, comorbidities, long latency and different challenges being present at different times [45].

Self-management resources empower cancer survivors to manage their healthcare needs [43, 44]. Iterative discussions with the advisory group and empirical data confirmed self-management as suitable for social eating support. It aligns with the chronicity of social eating challenges [33, 34] and incorporates IFSMT concepts like confidence and motivation [29]. The intervention integrates environmental and context-specific elements, suggesting its potential for other HNC survivorship challenges like lymphedema [46], communication [47], intimacy [48] and relationships [49].

Mental health challenges from HNC treatment [50] and reduced social eating highlight the urgency of addressing this issue. The intervention embeds a sensitive approach to the psychological difficulties associated with social eating. Improved QOL and lower depression levels are linked to fewer social eating challenges [51–55], suggesting social eating interventions could serve as primary prevention measures for mental health and psychosocial QOL indicators [56, 57].

## Clinical implications

The key clinical implication of the ‘Eating with Others’ intervention is the availability of a novel, evidence-based self-management guide that HCPs can integrate into routine practice. This guide addresses one of the most common and persistent impacts of HNC treatment, aiming to improve the QOL for both patients and their FMs. Holistic needs assessments in HNC survivorship care should address food and drink concerns, including the social dimension of eating [58]. Evaluating social eating needs and challenges at various intervals is essential due to their dynamic nature and changing importance, thus minimising barriers to support [59]. Whilst prehabilitation may initiate social eating support, research and reproducible services are limited [60–62].

HCPs should personalise information based on individual needs, preferences, context and environment [63, 64]. Tailored information should consider how often patients eat with others, with whom, family members they live with and their role in social eating [65, 66]. Assessments should include both patients and FMs, encouraging the integration of family-centred practices. This approach fosters an environment where FMs can openly share their experiences and challenges related to social eating, thus providing comprehensive support. By offering practical tools and strategies, the intervention ensures that both patients and their families receive relevant and effective support, ultimately enhancing the overall quality of survivorship care.

## Further research

The next phase, guided by the PBA framework, is to determine feasibility and effectiveness within clinical practice [24]. Additional research is needed to explore social eating with patients 5 + years beyond HNC and cultural influences on motivation, confidence and habits of social eating for HNC patients, including international comparisons and traditions.

Consideration should be given to the delivery platform for this intervention, especially for patients of different ages, genders, backgrounds and preferences. In the evolving landscape of healthcare, with the rise of digital healthcare, thoughtful consideration should be given to digitally delivering this intervention [67].

Although focused on HNC patients, social eating challenges are documented across various patient populations, such as those with inflammatory bowel disease [68], gastroparesis [69], oesophageal cancer [70], stroke [71], intellectual disabilities [72], autism [73], cerebral palsy [74] and dementia [75]. Physical, psychological and social factors may impede participation or enjoyment of social eating, especially in palliative care and cancers affecting children and young people [66]. The principles from this research may apply to other groups facing social eating difficulties.

## Limitations

One of the limitations of this research continued to be the disruption of COVID-19 and the impact it had on recruiting from participating healthcare trusts. The ongoing impact on a person’s ability to eat with others was inhibited during this period, and the interviews during Phase 1 (patient and FMs) were quite frequently reliant on memory. Therefore, it is not known if the persistent delays in the ability of patients to eat with others due to COVID-19 instilled avoidance habits. Further testing should occur with additional stakeholders such as FMs and more patients, despite the final participants in cycle 3 having no significant updates to the ‘Eating with Others’ prototype; this may not be representative of the full HNC hypernym.

## Conclusion

This novel ‘Eating with Others’ intervention, underpinned by our study’s empirical research, the principles of Engel’s [24] biopsychosocial model and the IFSMT [27] and guided by the PBA [22], resulted in a patient-centred, self-management and family-supported resource to address persistent challenges in social eating among HNC survivors. The successful integration of these pertinent theoretical frameworks, alongside positive feedback from the advisory groups and qualitative testing, underscores the intervention’s potential effectiveness and acceptability. It also positions this ‘Eating with Others’ intervention as a potentially valuable and comprehensive resource for HNC patients and their families to help them successfully navigate social eating challenges.

## Data Availability

No data sets were generated or analysed during the current study.
